# Assessing the quality and communicative aspects of patient decision aids for early-stage breast cancer treatment: a systematic review

**DOI:** 10.1007/s10549-019-05351-4

**Published:** 2019-07-24

**Authors:** Ruben Vromans, Kim Tenfelde, Steffen Pauws, Mies van Eenbergen, Ingeborg Mares-Engelberts, Galina Velikova, Lonneke van de Poll-Franse, Emiel Krahmer

**Affiliations:** 1 Department of Communication and Cognition, Tilburg University, Warandelaan 2, 5035 AB Tilburg, The Netherlands; 2 Chronic Disease Management, Philips Research, Eindhoven, The Netherlands; 3 Department of Research, Netherlands Comprehensive Cancer Organization (IKNL), Utrecht, The Netherlands; 4 Department of Medical Ethics and Philosophy of Medicine, Erasmus MC, Rotterdam, The Netherlands; 5 Department of Surgery, Sint Franciscus Vlietland Group, Rotterdam, The Netherlands; 6 Leeds Institute of Medical Research at St Jame’s, University of Leeds, Leeds, UK; 7 Division of Psychosocial Research & Epidemiology, The Netherlands Cancer Institute, Amsterdam, The Netherlands; 8 Department of Medical and Clinical Psychology, Tilburg University, Tilburg, The Netherlands

**Keywords:** Breast cancer, Decision aids, Patient education, Risk communication, Shared decision-making, Treatment decision-making

## Abstract

**Purpose:**

Decision aids (DAs) support patients in shared decision-making by providing balanced evidence-based treatment information and eliciting patients’ preferences. The purpose of this systematic review was to assess the quality and communicative aspects of DAs for women diagnosed with early-stage breast cancer.

**Methods:**

Twenty-one currently available patient DAs were identified through both published literature (MEDLINE, Embase, CINAHL, CENTRAL, and PsycINFO) and online sources. The DAs were reviewed for their quality by using the International Patient Decision Aid Standards (IPDAS) checklist, and subsequently assessed to what extent they paid attention to various communicative aspects, including (i) information presentation, (ii) personalization, (iii) interaction, (iv) information control, (v) accessibility, (vi) suitability, and (vii) source of information.

**Results:**

The quality of the DAs varied substantially, with many failing to comply with all components of the IPDAS criteria (mean IPDAS score = 64%, range 31–92%). Five aids (24%) did not include any probability information, 10 (48%) presented multimodal descriptions of outcome probabilities (combining words, numbers, and visual aids), and only 2 (10%) provided personalized treatment outcomes based on patients and tumor characteristics. About half (12; 57%) used interaction methods for eliciting patients’ preferences, 16 (76%) were too lengthy, and 5 (24%) were not fully accessible.

**Conclusions:**

In addition to the limited adherence to the IPDAS checklist, our findings suggest that communicative aspects receive even less attention. Future patient DA developments for breast cancer treatment should include communicative aspects that could influence the uptake of DAs in daily clinical practice.

**Electronic supplementary material:**

The online version of this article (10.1007/s10549-019-05351-4) contains supplementary material, which is available to authorized users.

## Introduction

In early breast cancer care, there has been rapid growth in the development of patient decision aids (DAs) to support the process of shared decision-making (SDM) between patients and their clinician [1]. DAs are tools (aimed at patients and distributed by clinicians) that provide information about treatment options and associated risks of side-effects and disease recurrence, and help patients clarify their values and preferences [2, 3]. Moreover, DAs should encourage patients to (actively) participate in the SDM process with their clinician [3, 4]. Despite great promise and the increasing interest in developing DAs [1, 2], the extent to which they are implemented into daily clinical practice appears to be limited [5, 6].

One reason for this might be the variability in the characteristics and quality of DAs for early breast cancer treatment [7]. Assessing the quality of DAs (e.g., whether the DAs’ content is reliable and evidence-based, or how they were developed and field-tested) is relevant to patients and clinicians [8], since a lack of trust in or familiarity with the quality of DAs could explain why clinicians do not distribute them to their patients [9]. Typically, the validated international patient decision aids standards (IPDAS) checklist is used to ensure the quality of DAs [10], and covers a variety of dimensions, ranging from information about treatment options and outcome probabilities to decision guidance and development process. Although the IPDAS is considered the gold standard for developing and evaluating DAs [11], being IPDAS compliant does not guarantee that DAs will reach the hands of patients.

We argue that another factor is the extent to which DAs pay attention to the *communicative aspects*. In fact, DAs include many communication aspects that may influence the use and understanding of the tools by patients and clinicians, but are not covered by the IPDAS checklist [12]. These include, for instance, how DAs *present information* about treatment options and associated outcome probabilities to patients (e.g., only words or numbers, or in combination with visual aids) [13], or how they communicate uncertainty around statistics. Another communicative aspect is how DAs *interact* with patients to elicit their values or preferences (e.g., value-clarification exercise) [14], or to provide patients with *personalized information* based on their personal and tumor characteristics (e.g., personalized risk or survival estimates), all of which can improve patient and clinician’s understanding of the personal and clinical situation at hand. Furthermore, aspects like the *suitability* (e.g., complex language use), *accessibility* (e.g., only internet-based), or *source of information* (e.g., reliable outcome probabilities) could disturb the communication process between the DA, patient, and clinician [15]. All these aspects are important elements of the communication process [16], and DAs that pay less attention to these aspects may limit their ability to be distributed by clinicians and to be used and/or comprehended by patients.

Although some reviews have shown the effectiveness of DAs in early breast cancer care [1, 17, 18], there has been no review on the quality and use of communicative aspects among existing DAs for patients facing early breast cancer treatment decisions. Therefore, the aims of this systematic review were (1) to make an inventory of currently available patient DAs for early-stage breast cancer treatment in both English and Dutch, (2) to critically review their quality based on the IPDAS criteria, and (3) to assess to what extent they pay attention to various communicative aspects.

## Methods

This systematic review is conducted and reported in compliance with the Preferred Reporting Items for Systematic Reviews and Meta-Analyses (PRISMA) guidelines [19].

### Data sources and search strategy

A systematic search of both published literature and online sources was conducted to identify and obtain DAs for patients facing early breast cancer treatment decisions. To obtain DAs with associated studies through *published literature*, we searched the following databases: MEDLINE (via PubMed), EMBASE, Cochrane Library, The Cumulative Index to Nursing and Allied Health Literature (CINAHL), and PsycINFO. Given that the IPDAS checklist was launched in 2006, we searched the databases from January 2006 until March 2018. Reference lists and author names were searched to identify additional publications that met the eligibility criteria. The search strategy included a combination of keywords, synonyms, and MeSH headings relating to the concepts of breast cancer, DAs, SDM, and treatments (Supplementary Material 1). To obtain DAs without associated studies through *online sources*, we searched the Ottowa Decision Aid Library Inventory (https://decisionaid.ohri.ca/cochinvent.php), and Google^TM^ (search terms “decision aid,” “breast cancer,” and “treatment”) in both Dutch and English for which the first 100 hits were analyzed.

### Inclusion and exclusion criteria

We developed inclusion and exclusion criteria for the identification of scientific studies and for decision aids. For the *studies* obtained through published literature, the inclusion criteria include those that were (1) reported in a scientific journal (peer-reviewed); (2) published between 2006 and 2018; (3) written in English or Dutch. Study types eligible for inclusion were (1) (non-)randomized controlled trials or experimental studies that addressed the impact of DAs as intervention on decisional outcomes or treatment choice; (2) development and/or evaluation of the DAs (e.g., protocol, developmental, evaluation, usability testing, or observational studies). Target populations of studies included newly diagnosed patients with early-stage breast cancer facing treatment decision-making.

For both *DAs* obtained through published literature and online sources, the following exclusion criteria applied: DAs (1) developed for women with advanced stages of breast cancer or for breast cancer screening; (2) in the format of predictive or decision-support tools (e.g., Predict-UK, Adjuvant!Online) since such tools are aimed for both clinician and patients; (3) in the format of phone calls, online support groups, interviews, nomograms, or audiotapes, since such formats could not be analyzed. Finally, the following inclusion criteria applied: DAs that were (1) published between 2006 and 2018; (2) (publicly) available; (3) fully accessible (e.g., no monetary costs associated with the DA such as one time purchase, or no need to be prescribed by a certain healthcare system or clinician); (4) written in English or Dutch.

### Study and decision aid selection

Two reviewers (RV, KT) screened all retrieved articles for relevance based on title and abstract for initial eligibility. The overall kappa score for inter-rate agreement during the screening phase was strong (κ = 0.97) [20]. Afterwards, the few disagreements were resolved through discussion or adjudication by a third person. Subsequently, the same two reviewers independently evaluated the articles that passed the previous screening phase based on the eligibility criteria and disagreements were resolved through discussion and consensus between the two reviewers. The overall kappa score during the study eligibility phase was strong (κ = 0.91). Data extraction of the included studies and DAs were independently assessed by two reviewers.

### Assessment of decision aids

The assessment of the identified DAs consisted of two parts. DAs were first reviewed for their quality according to IPDAS criteria, after which they were critically assessed on a communicative aspect checklist. Each DA was independently assessed by two coders (four coding teams in total). Inter-rate agreements (κ) achieved by the teams ranged from 0.74 to 0.86 for the IPDAS checklist (mean κ = 0.81), and from 0.76 to 0.90 for the assessment of CAs (mean κ = 0.83). The total, average inter-rate agreement was good (κ = 0.82).

#### Quality of decision aids

Quality of the included DAs was assessed by using the IPDAS Collaboration criteria framework. The IPDAS instrument (Supplementary Material 2) [10] consists of 36 items divided into eight dimensions: (i) *information about options* (items 1–8), (ii) *outcome probabilities* (items 9–16), (iii) *clarifying values* (items 17–20), (iv) *decision guidance* (items (21–22), (v) *development process* (items 23–28), (vi) *using evidence* (items 29–33), (vii) *disclosure and transparency* (items 34–35), and (viii) *plain language* (item 36). Since not all DAs had been evaluated in scientific studies, we decided to exclude the two items related to the evaluation dimension. Response options for each criteria item were ‘yes’ and ‘no’ (coded as 1 and 0, respectively). For each DA, the number of IPDAS items met was converted to percentages of the total number of items.

#### Communicative aspects of decision aids

The use of communicative aspects by the DAs was assessed by a recently developed and validated communicative aspect checklist for patient DA (Supplementary Material 3) [12]. This tumor-independent checklist consists of 76 items divided into seven CAs: (i) *information presentation* (items 1–26), (ii) *information control* (items 27–33), (iii) *personalization* (items 34–40), (iv) *interaction* (items 41–55), (v) *accessibility of information* (items 56–64), (vi) *suitability of information* (65–68), and (vii) *source of information* (items 69–76). Response options for each item were ‘yes’ and ‘no’ (coded as 1 and 0, respectively; seven items needed to be recoded). Since six items were only applicable to web-based DA, the total number of items for paper-based DAs was 70, and for web-based 76. For each DA, the number of communicative aspect items met was converted to percentages of the total number of items. Note that a higher communicative aspects score does not necessarily indicate a higher quality DA; it only suggests that more items from the communicative aspects checklist were taken into consideration.

## Results

### Search results and decision aid characteristics

**Fig. 1:**
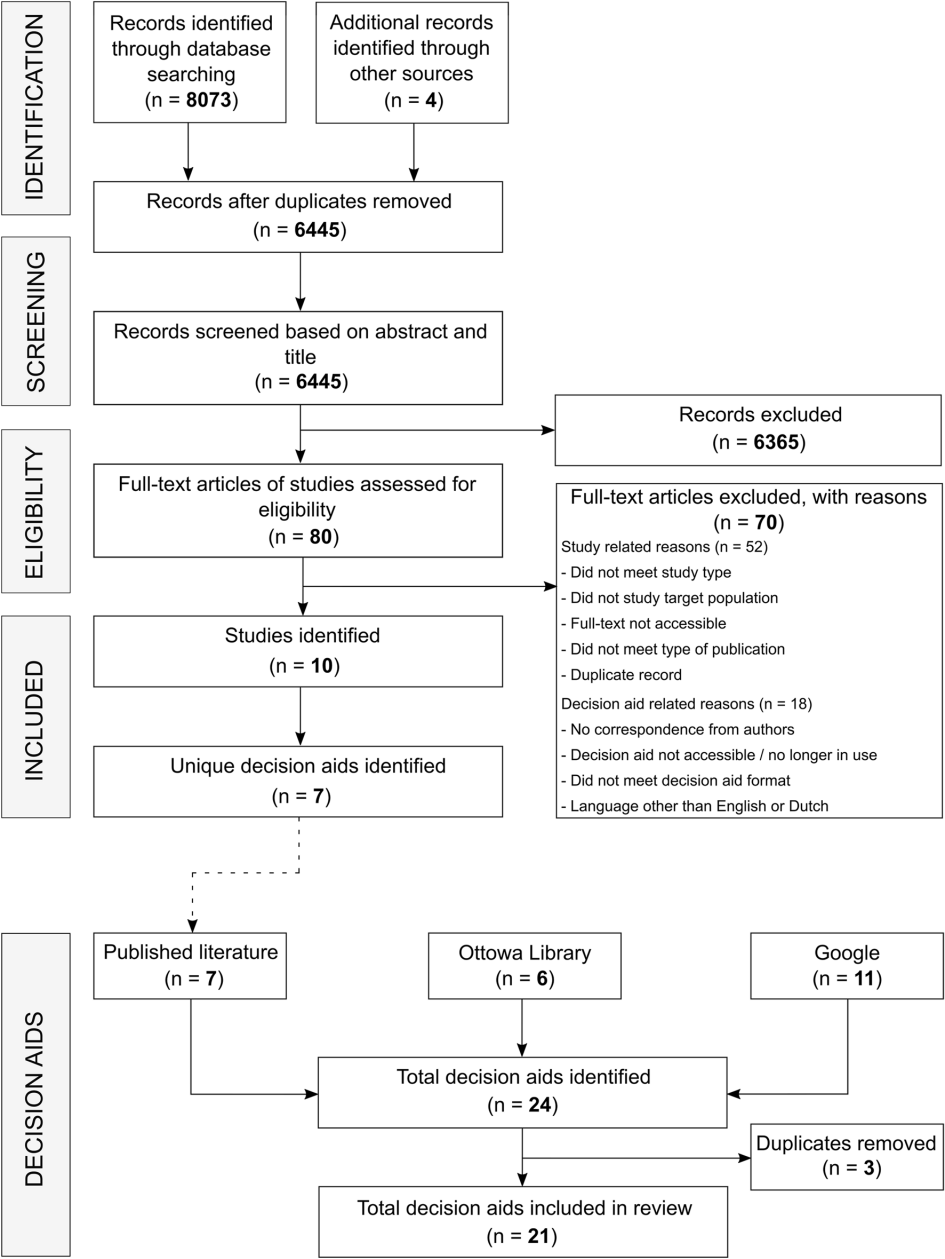
Flowchart of study and decision aid selection process

In total, 8073 records were identified through five databases, and four additional records through other sources (Fig. 1). Screening titles, abstracts, and full-texts yielded ten eligible studies, including seven unique DAs. An additional search through online sources resulted in another 14 unique DAs, leading to a total of 21 DAs included in this review (Table 1). Ten aids originated from the United States, five from the Netherlands, five from Australia, and one from Canada. Eleven of the DAs were web based and ten were paper based. Most DAs discussed reconstruction surgery (11) and/or surgery (10; mastectomy vs. breast-conserving therapy) as treatment options, followed by (adjuvant) radiotherapy (9), systemic therapy (7; (neo)adjuvant chemotherapy and hormonal therapy), and lymph node surgery (3; axillary dissection and sentinel node biopsy). Year of last update ranged from 2008 to 2018, but most (13) had been updated in 2017 or 2018. Seven DAs had 1 or more associated studies [21–30] of which three were RCTs, five evaluation and/or development studies, and two protocol studies (Table 2).

**Table 1:** Summary of the decision aids included in the systematic review

ID	Title	Organization/authors	Country	Treatment discussed	Last update	Format
1	Mastectomy or breast-conserving therapy	Harwood (2011)	AUS	BCS; mastectomy	Aug 2008	Paper
2	Axillary dissection or a sentinel node biopsy	Harwoord (2011)	AUS	Lymph node surgery	Aug 2008	Paper
3	Understanding ductal carcinoma in situ (DCIS) and deciding about treatment	National breast and ovarian cancer center	AUS	BCS; mastectomy; radiotherapy; hormonal therapy	2010	Paper
4	Frankly speaking about cancer: breast reconstruction	Cancer support community	USA	Reconstruction surgery	2012	Paper
5	Surgery choices for women with DCIS or breast cancer	National cancer institute	USA	BCS; mastectomy; reconstruction surgery; lymph node surgery	Nov 2012	Paper
6	A guide for women who are considering breast cancer treatment with chemotherapy and/or hormonal therapy before surgery	Australia and new zealand breast cancer trial group (ANZBCTG) Zdenkowski (2016, 2018)	AUS	(Neo)adjuvant chemotherapy; (Neo)adjuvant hormonal therapy	Dec 2014	Paper
7	A patchwork of life: One woman’s story. For women making breast cancer treatment decisions	Dan L. Duncan comprehensive cancer center, Jabaja-Weiss (2006, 2011)	USA	BCS; mastectomy; reconstruction surgery; radiotherapy; hormonal therapy; chemotherapy	Aug 2015	Web
8	Early-stage breast cancer: Choosing your treatment	Health dialog	USA	BCS; mastectomy; reconstruction surgery; radiotherapy; lymph node surgery	Jul 2016	Paper
9	iCanDecide	Cancer surveillance and outcomes research team, University of Michigan, Hawley (2017a, 2017b, 2018)	USA	BCS; mastectomy; reconstruction surgery; radiotherapy; chemotherapy; hormonal therapy	2017	Web
10	Breast reconstruction: Is it right for you?	Health dialog	USA	Reconstruction surgery	Jul 2017	Paper
11	Keuzehulp borstkanker	PATIENT+	NL	BCS; mastectomy; radiotherapy	Aug 2017	Web
12	Keuzehulp borstreconstructie	PATIENT+	NL	Reconstruction surgery	Aug 2017	Web
13	Breast RECONstruction decision aid (BRECONDA)	Breast cancer network Australia, Westmead breast cancer institute, Macquarie University, Sherman (2016)	AUS	Reconstruction surgery	Oct 2017	Web
14	OPTIONS: what are my options for breast cancer treatment?	Wong (2011)	CAN	Radiotherapy; hormonal therapy	Oct 2017	Paper
15	Breast cancer surgery options	Allina Health	USA	BCS; mastectomy; radiotherapy	2018	Paper
16	Borstreconstructie keuzehulp	Zorgkeuzelab	NL	Reconstruction surgery	Jan 2018	Web
17	Breast cancer: should I have breast reconstruction after a mastectomy?	Healthwise	USA	Reconstruction surgery	Mar 2018	Web
18	Breast cancer: should I have chemotherapy for early-stage breast cancer?	Healthwise	USA	Chemotherapy	Mar 2018	Web
19	Breast cancer: should I have breast-conserving surgery or a mastectomy for early-stage breast cancer?	Healthwise	USA	BCS; mastectomy	Mar 2018	Web
20	Borstkanker keuzehulp	Zorgkeuzelab Maastricht UMC+	NL	BCS; mastectomy; reconstruction surgery; radiotherapy; chemotherapy	Oct 2018	Web
21	Borstkanker RAdiotherapie SAmen beslissen (BRASA)	MAASTRO Clinic, Maastricht University, Netherlands Cancer Institute	NL	Radiotherapy	Oct 2018	Web

*BCS* breast-conserving surgery

**Table 2:** Summary of the studies included in the systematic review

DA ID	First author, year	Country	Study design	Study population	Methods	Results
1,2	Harwood, 2011	AUS	Development/Evaluation and pilot study	Development/Evaluation study: women who had already had surgery for breast cancer (stages I and II, *n * = 28) Pilot study: newly diagnosed patients with early breast cancer (stages not mentioned, *n * = 11)	There were two phases of this study. The first phase involved patients evaluating the two DAs, and the second phase involved determining the effectiveness of the DAs. During both phases, study outcomes were treatment chosen, patient knowledge, decisional conflict, and satisfaction with decision-making.	Patients in the historical control group reported positive feedback on the DAs, and patients in the intervention pilot group found the DAs to be helpful. Results from the pilot study suggested a possible reduction in decisional conflict, and increase in decisional satisfaction, knowledge, and choice of axillary clearance (instead of sentinel node biopsy) in the intervention pilot group.
6	Zdenkowski, 2016	AUS	Development/protocol evaluation study	Newly diagnosed patients with invasive and operable breast cancer (target *n * = 50)	A pre-post design will be used to evaluate the acceptability and feasibility of the DA. Primary outcomes will be acceptability and feasibility, and secondary outcomes will be decision conflict, knowledge, information and involvement preference, agreement between preferred and achieved decision.	N.A.
6	Zdenkowski, 2018	AUS	Evaluation study (pre-post design)	Newly diagnosed patients with operable invasive breast cancer (*n * = 59)	Patients first completed a baseline questionnaire (test 1), subsequently received the DA prior to consultation, and then completed a follow-up questionnaire after consultation (test 2), before surgery (test 3) and 12 months after registration (test 4). Study outcomes: as above.	The DA was found to be feasible (with most patients having accessing it) and acceptable (with the majority of the patients seeing the DAs as useful for their decision about treatment). Moreover, post-DA, decisional conflict, anxiety, and distress decreased significantly.
7	Jibaja-Weiss, 2006	USA	Evaluation study	Newly diagnosed patients with early breast cancer (stages I–IIIA, *n * = 51)	Patients answered a number of questions after diagnosis, and after completing the DA. Study outcomes were patients’ use of the values clarification exercise, perceived clarity of values, and decision conflict scores (low literacy version).	Over half of the participants performed the values clarification exercise. The use of the DA was associated with lower levels of decisional conflict (compared to baseline scores) and lower levels of feeling unclear about values.
7	Jibaja-Weiss, 2011	USA	RCT	Newly diagnosed patients with early breast cancer (stages I–IIIA, *n * = 76)	Patients were randomized to either the intervention group (DA plus usual care) or the control group (usual care only). Study outcomes were treatment preference, breast cancer knowledge, satisfaction with decision, satisfaction with decision-making process, and decision conflict (low literacy version).	Patients who received the DA were more likely to indicate a preference for mastectomy rather than breast-conserving surgery, were more knowledgeable and clearer about their values compared to the control group. No differences were found in satisfaction with the decision or the decision-making process between the two groups.
9	Hawley, 2016	USA	Evaluation and pilot study	Newly diagnosed patients with early breast cancer (stage 0, I, or II, *n * = 101)	Patients were randomized to either the intervention group (who viewed the DA first) or the control group (who took a survey prior to viewing the DA). Study outcomes for the evaluation were knowledge (about treatment options and breast cancer) and decisional appraisal.	Patients who viewed the DA first had higher scores on decisional appraisal than the control group. However, no statistically significant differences were found in knowledge about treatment options between the two groups.
9	Hawley, 2017	USA	RCT protocol	Newly diagnosed patients with early breast cancer (DCIS, or stage I–II, target *n *= 222 per arm)	A two-arm RCT will be conducted to evaluate the impact of a tailored DA (intervention group) on decision quality, decision satisfaction, deliberation, and decision preparedness (as primary study outcomes) compared to the same non-tailored static DA (control group).	N.A.
9	Hawley, 2018	USA	RCT	Newly diagnosed patients with early breast cancer (stage I–II, *n * = 496)	Patients were randomly allocated to the intervention group (tailored DA) or control group (non-tailored static DA). Primary study outcome was high-quality decision-making (which consisted of (1) knowledge about risks and benefits of treatment options and (2) values-concordant treatment).	The use of a tailored DA was positively associated with high-quality decisions compared to using a non-tailored DA. Furthermore, patients in the intervention group had higher levels of knowledge than the control group. However, no differences were found in values-concordant treatment decisions between the two groups.
13	Sherman, 2016	AUS	RCT	Newly diagnosed patients with early breast cancer or ductal carcinoma in situ (DCIS, stages I–III, *n *= 222)	An RCT was conducted to determine the effectiveness of a DA for deciding whether to have breast reconstruction or not. Patients were randomized to either the intervention group (DA + plus standard information) or the control group (standard information). Study outcomes were decisional conflict, satisfaction with information, and decisional regret (1 and 6 months after exposure).	At both 1- and 6-month follow-up, the use of the DA was associated with lower levels of decisional conflict and higher levels of satisfaction with the information compared to the control group. There were no differences in decisional regret between the two groups.
14	Wong, 2011	CAN	Development and evaluation study	Development study: patients with early breast cancer who had already had radiotherapy (stage I, *n * = 12) Evaluation study: Newly diagnosed patients with early breast cancer (stage I, *n *= 36)	There were two pilot studies in this study. The first involved the development of the DA in which patients were asked to review the acceptability of the aid. The second pilot was a pre-post test aimed at examining the effectiveness of the DA on decisional conflict, knowledge, impact of event, and treatment choice.	The majority of patients rated the DA as (extremely) satisfied. In comparison to the baseline scores (pre-test), patients experienced less decisional conflict and were more knowledgeable after using the DA (post-test).

*DA* decision aid, *N.A.* not applicable, *RCT* randomized controlled trial

### Quality of decision aids

**Fig. 2:**
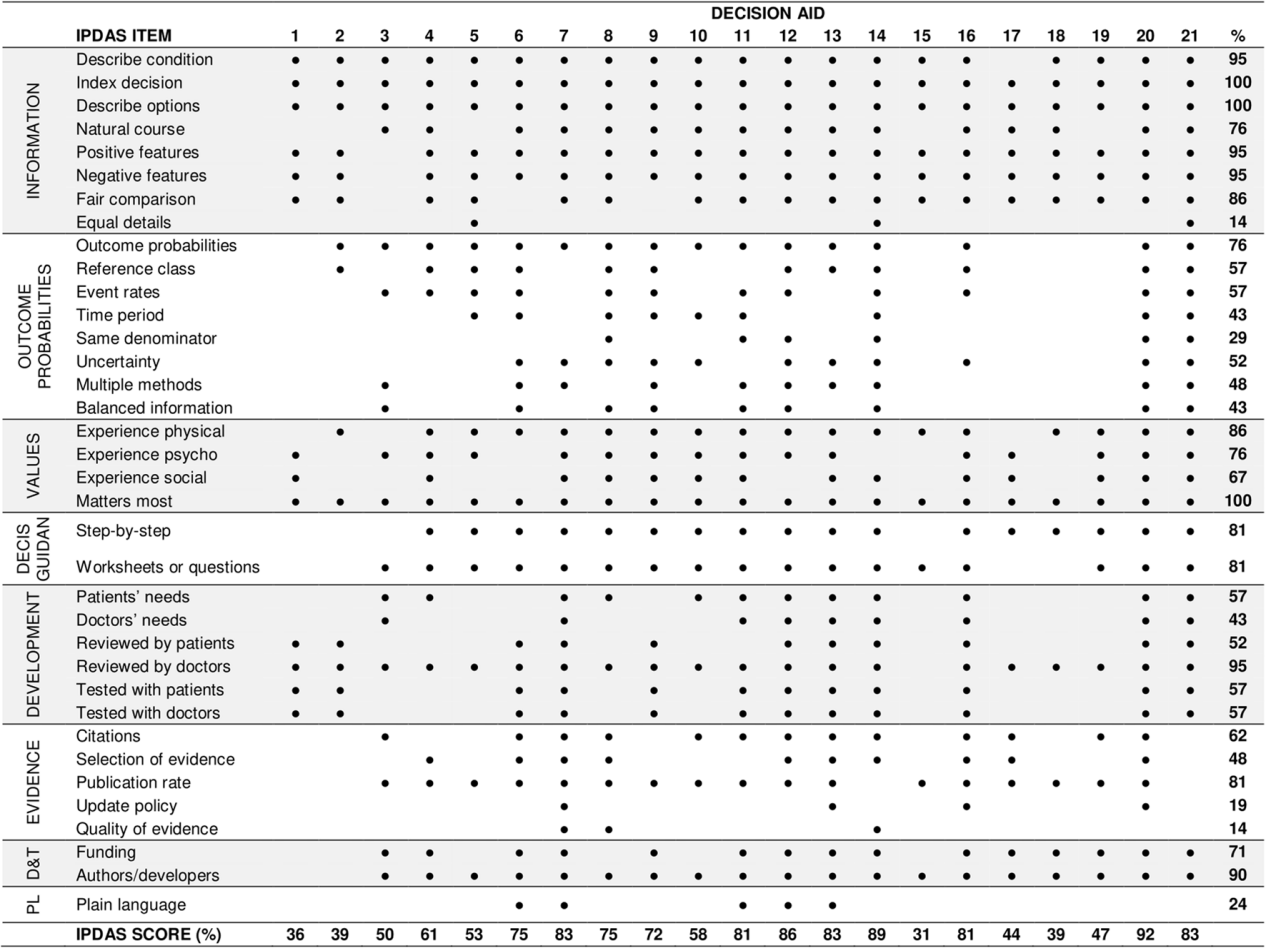
The international patient decision aid standard (IPDAS) scores for each decision aid. *Decis guidan* decision guidance, *D&T* disclosure and transparency, *PL* plain language

**Fig. 3:**
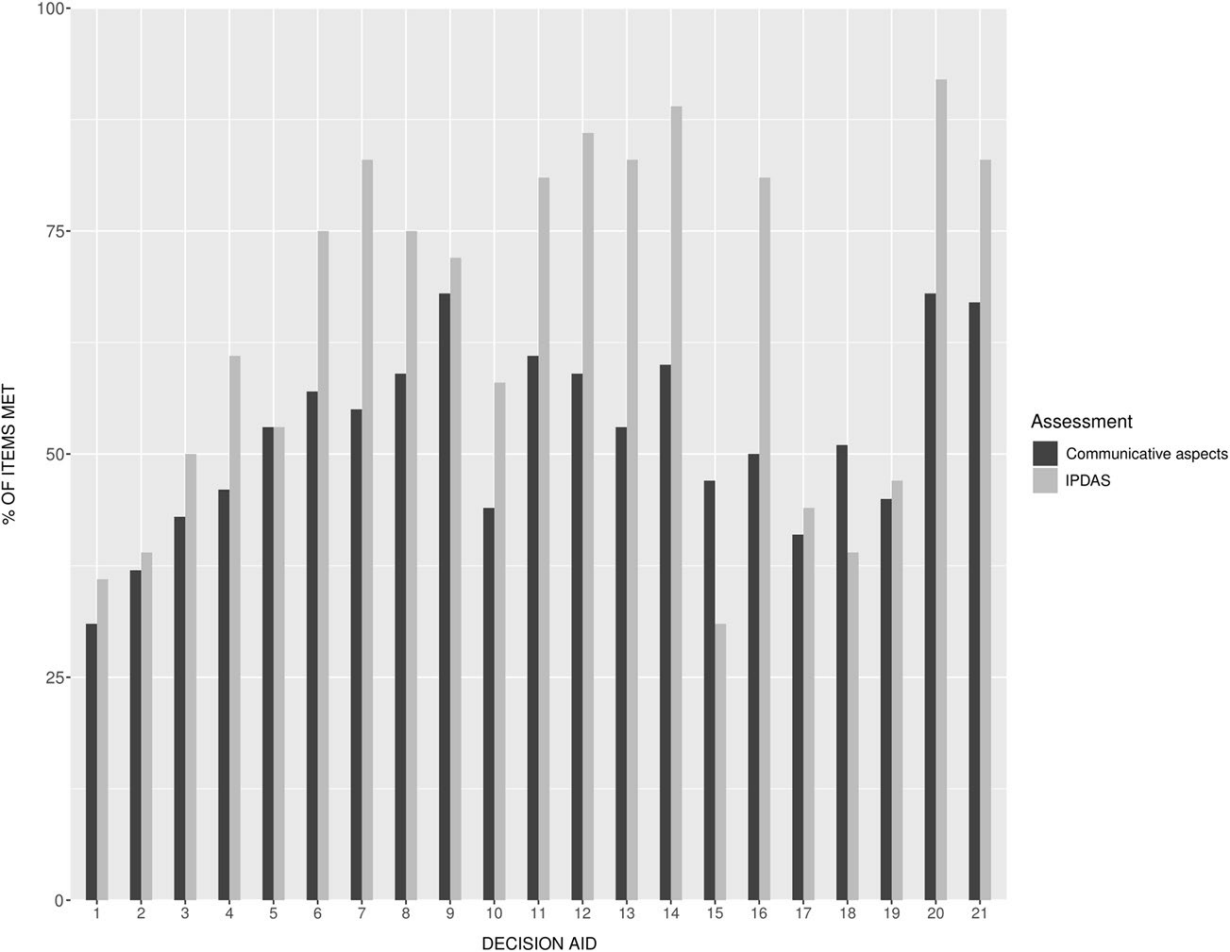
Percentage of items met on the IPDAS and communicative aspects checklist for each decision aid. Decision aids are presented in chronological order (based on year of last update)

None of the DAs met all of the IPDAS criteria, and the total percentage of IPDAS criteria met by the DAs ranged from 31 to 92% (mean IPDAS score (*M*) = 64%, standard deviation (SD = 20%), see Fig. 2). The seven DAs with associated studies had slightly higher IPDAS scores (*M *= 68%, SD = 8%) than DAs without associated studies (*M *= 63%, SD = 5%). The best performing DAs on the IPDAS checklist were DA12, DA14, and DA20 (Fig. 3).

Most aids showed high performance on the dimensions information about treatment options, clarifying values, disclosure and transparency, and decision guidance. For instance, all DAs (100%) presented the available treatment options, with the majority of them explaining both positive and negative features of the options (95%). All aids asked patients to think about positive and negative features of the options that matter most to them (100%). Mixed performance was observed for items related to evidence, development process, and outcome probabilities. For instance, as mentioned by the DA or associated paper, almost all aids were reviewed by doctors (95%), but only half of them were reviewed by (52%) or tested with (57%) patients. Five aids (24%) did not contain any outcome probabilities. Of the aids that did contain probability information, many did not adhere to good practice guidance on communicating essential elements such as providing event rates (57%), keeping the same denominators (29%), reporting time period (43%), or uncertainty (52%). Moreover, only four DAs (19%) reported the update policy and three (14%) discussed the quality of the evidence used. Finally, regarding the dimension of plain language, only five aids (24%) reported acceptable readability levels (e.g., 8th–10th grade (Flesch-Kincaid) reading level).

### Communicative aspects of decision aids

**Fig. 4:**
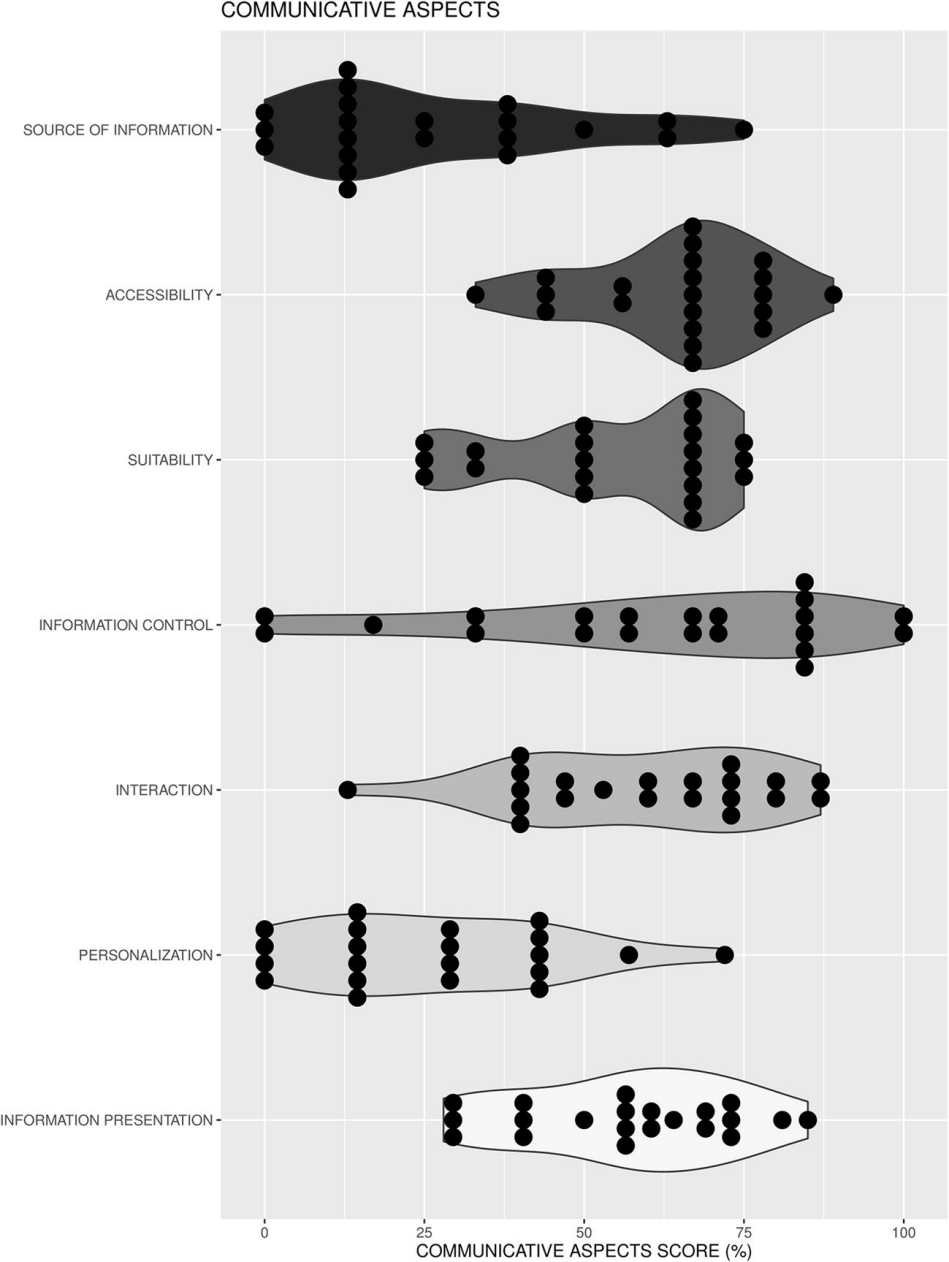
Violin plots of the percentage of items met on the communicative aspects checklist separated for each aspect. For each violin plot, dark dots represent the DAs

A full summary of the results on the assessment of communicative aspects can be found in Supplementary Material 3. The overall percentage of communicative aspect items met by the DAs ranged from 31% to 68% (*M *= 52%, SD = 10%). The seven DAs with associated studies had similar communicative aspects scores (*M *= 52%, SD = 5%) compared to DAs without associated studies (*M *=52%, SD = 2%). The best performing DAs on the communicative aspects checklist were DA9, DA20, and DA21 (Fig. 3). In general, the majority of the aids met most items related to accessibility; mixed results were found for items with respect to information presentation, information control, interaction, and suitability of information; the least number of items met was shown for personalization and source of information (Fig. 4).

#### Information presentation

All DAs used different presentation formats for communicating outcome probabilities. Of the aids, 3 (14%) did not use any method, 2 (10%) used words-only (e.g., verbal descriptions), 6 (29%) used a combination of words and numbers, and 10 (47%) applied a combination of words, numbers, and visuals. Of the 16 aids that used numerical methods, natural frequencies were most often used (12; 75%) followed by percentages (11; 69%); for the 10 aids that used visual methods, icon arrays were the most common (9; 90%), followed by a pie chart or line graph (both 1; 10%). Of the 18 aids that communicated probability information, 14 (78%) described uncertainties around them, typically with verbal methods (13; 93%), followed by numerical ranges (8; 57%), and visually presented confidence intervals (1; 7%). Variations were also observed in presenting disease-related information (6 used text-only, 10 a combination of text and visual/audiovisual), and procedures of treatments (6 text-only, 15 a combination of text and visual/audiovisual). Finally, a significant number of DAs (19; 90%) presented information in an unbalanced way; 9 aids (43%) used more space/text for a specific treatment option, the majority provided an unequal number of positive (12; 55%) and negative features (17; 85%) across the treatment options, and of the 16 aids that included statistical information only 5 (31%) displayed such statistics in a similar way for each option.

#### Personalization

The majority of the DAs (14; 67%) were tailored towards the breast cancer stage (e.g., early-stage). However, tailoring towards the type of treatment (7; 33%), specific populations (3; 14%), or other breast cancer-related factors (4; 19%) (e.g., HER2 status) occurred less frequently. Five aids (24%) allowed patients to tailor the content of the DA, 3 (14%) to tailor information to patients’ own preference for the mode of information presentation, and only 2 DAs (10%) allowed patients to view individualized outcome probabilities based on their own situation.

#### Interaction

Several interaction methods had been used by the DAs. For comparing treatment options (20; 95%), most used side-by-side tables or verbal comparisons (both 17; 85%), 6 (30%) included ranking or rating exercises, and 2 (10%) applied conjoint analysis/visual analogue scales based on patients’ preferences. For clarifying patients’ values, the majority (20; 95%) passively asked patients to think about their personal values, and about half used active methods such as weighting exercises (12; 60%) and/or sliders to assign values to preferences (9; 45%). Feedback was also given in different ways. Twelve aids (57%) showed the progress of the aid, 12 (57%) provided a summary of patients’ values and preferences, 17 (81%) included a print option. About half (10; 48%) provided space for note taking, and 8 (38%) included a knowledge test.

#### Information control

Nine aids (43%) allowed patients to only receive information that they wanted to read. The majority (18; 86%) provided a step-by-step way to move through the DA, and 16 (76%) gave patients the opportunity to read more about a specific topic of interest. Only 5 aids (24%) allowed for patients to search for specific keywords or topics in the aid.

#### Accessibility and suitability

Regarding the suitability of information, almost all DAs (19; 90%) used a conversational (writing) style, and only 6 (29%) contained irrelevant illustrations that did not have any link with the messages being presented. Of the aids that included audiovisual material, only 1 (17%) had videos of less than 1 min. Most aids (16; 76%) were lengthy and contained more than ten (web) pages. Regarding accessibility of the aids, 16 (76%) were freely available on the web, and 5 (24%) required a login code to get full access. Thirteen DAs (62%) reported the date of last update, but only 2 (10%) reported the update frequency. All except for 1 aid could be used on multiple devices such as a laptop or smartphone, or were self-administered. Six aids (29%) required staff assistance in order to start with the aid.

#### Source of information

Of the 18 DAs that communicated outcome probabilities, most included probabilities for treatment side-effects (12; 67%), followed by recurrence of cancer (12; 67%). Numerical information related to survival rates (4; 22%) or quality of life outcomes (5; 28%) occurred less frequently. Only 5 DAs (28%) reported the original source of the probabilities (e.g., RCTs or population-based data), of which 3 (60%) provided detailed information about the patients included in the data (sets) and 1 (20%) about the period of data collection.

## Discussion

In this systematic review, we identified 21 currently available patient DAs for early-stage breast cancer treatment, and critically reviewed their quality (as assessed by the IPDAS checklist [10]) and use of communicative aspects (as assessed by a communicative aspect checklist [12]). This review shows substantial variability in the quality of the DAs, with no existing DA meeting all of the internationally agreed IPDAS criteria. Many did not adhere to good practice guidance on providing information about the development, evidence used for the content, or reporting readability levels. This limited adherence to the quality criteria has also been found among existing DAs for patients with localized prostate cancer [7, 12]. Nevertheless, it is promising to see that most of the recently launched or updated DAs in our review (i.e., from 2017 onwards) have shown increased adherence to the IPDAS criteria (see Fig. 3), which suggests that current DA developers and/or clinicians are now taking these criteria much more into account than in the past. At the same time, however, patients can still easily find and make use of existing low-quality DAs, which may foster low implementation rates [5, 6].

We also observed that few DAs presented a thorough description of outcome probabilities of treatment options. In fact, three aids did not contain any probability information at all, and two only used verbal descriptions. Ideally, treatment decision-making is, among other elements such as patients’ preferences, guided by evidence-based probabilities of treatment outcomes such as survival rates, side-effects, or quality of life after treatment [3, 13]. Following the IPDAS guidelines, such outcomes may help newly diagnosed cancer patients in balancing the risks and benefits of options together with their clinician, and should therefore be incorporated in DAs [31]. Moreover, from an ethical point of view, patients should be fully and adequately informed^3^, and thus they should also be informed about outcome probabilities and their original sources [32]. The lack of statistical information for breast cancer DAs is remarkable and in contrast with DAs evaluated for men with localized prostate cancer of which all (except for one DA) contained numeric estimates regarding survival rates and side-effects of treatments [12].

The DAs that *did* communicate probability information showed great variability in *how* they communicated such statistical information. Most aids used numeric estimates such as natural frequencies or percentages, and only a few used visual aids such as icon arrays. However, several studies have shown that patients (especially with low numeracy skills) often misunderstand such statistics [33], especially when only being communicated in words [34]. Adding numbers in combination with visual aids may facilitate patients’ understanding of probabilities and overcome several biases such as denominator neglect or framing effects [13]. This multimodal strategy (e.g., using both words and pictures) is also useful for communicating other treatment information (e.g., procedures of treatments), which may lead to better information recall by patients [35]. Over the years, several best practices in the communication of evidence-based outcome probabilities have been developed [13, 33], and it is important that DA developers and clinicians who are communicating statistical information to patients are taking these sets of guiding principles into account.

One of the more significant communicative issues found in the reviewed DAs for early breast cancer concerns the lack of *personalization*. For instance, all (except for two) DAs communicated average outcome probabilities based on statistics of groups of prior patients, which may be difficult to apply to the situation of individual patients [36]. Clinical decision-support tools for explaining chemotherapy survival benefits exist (e.g., Predict-UK), and can already estimate personalized outcomes based on patients’ personal (e.g., age) and disease-related (e.g., tumor stage) characteristics entered by the clinician. However, such tools are often difficult to understand for patients and should always be used in consultation with a clinician. We therefore argue that patient DAs can be improved by incorporating patient-friendly versions (or result pages) of such personalized clinical prediction models into existing or novel DAs. However, a prerequisite for personalizing outcomes to individual patients is the availability of robust predictive models based on large amounts of clinical data [37, 38]. Recent technological advances in data science and artificial intelligence in combination with large population-based (e.g., cancer registries) or patient-reported outcome datasets offer promise for the generation of personalized treatment outcomes in DAs [12, 39].

This review further reveals some potential communicative issues of early breast cancer-specific DAs that could hinder their uptake in routine clinical practice. For instance, most aids provided extensive and detailed information about the options. This may be beneficial for patients who prefer detailed information about treatment options, but may discourage patients who do not have the need, time, or capacity for this [40]. Similarly, not all DAs were easily accessible for patients due to, for instance, limited access (i.e., login code), out-datedness of information, or poor findability. These accessibility issues might be barriers for especially patients with low literacy skills, who face difficulty in finding, evaluating, and obtaining online health information [41]. Next to that, clinicians may better appreciate the benefit of using and providing DAs to their patients if communicative aspects such as personalization (e.g., individualized treatment outcomes) or interaction (e.g., value-clarification exercises) are taken into account. Clinicians may wonder how a limited DA can add to their advisory consult and whether a low literacy patient can take advantage of this DA. It is plausible that improving these communicative aspects of DAs will lower the barrier for clinicians to distribute DAs to their patients.

Our review does have some limitations. First, most DAs were identified through online sources compared to the academic literature. Initially, we found 26 DAs with associated studies, which was comparable with the number of studies found by a related review [1]. In contrast with that review, we needed to have full access to the tools in order to accurately review their quality and communicative aspects. Hence, we could only obtain full access to a minority of those aids found through the academic sources. It should be noted, though, that this distribution of aids found via published literature or online sources is similar to distributions found in related reviews [7, 12], that used a similar method for identifying and reviewing the characteristics of DAs. Another limitation is that we could not link the IPDAS and communicative aspect scores to various SDM outcomes, mostly because of the lack of data. For instance, it may be that DAs that are personalized (in terms of content, amount of information, or mode of information delivery) are seen as more personally relevant and processed more deeply by patients [42]. The benefit of this in-depth processing is that patients may acquire better *knowledge* about their options, which makes them *better prepared* for their next consultation, with more time actively involved in a SDM process [43].

## Conclusion

SDM in early breast cancer care requires that patient and clinician are both well-informed about the clinical case and personal situation at hand. DAs have been developed to facilitate this process, but their implementation in routine clinical practice remains low. This review provides insights into the variability among currently available DAs for early breast cancer treatment, and shows that both their quality and use of various communicative aspects can be improved. In addition, even though adherence to the IPDAS checklist is important for ensuring high-quality DAs, our findings suggest that DA developers should also seriously consider communicative aspects that could influence the uptake of DAs in daily practice. Our results do not only have implications for clinicians who are involved in the development and use of DAs for breast cancer treatment, but also for clinicians outside of breast cancer who are facing similar complex and time-consuming clinical counseling scenarios with their patients.

## Electronic supplementary material

Below is the link to the electronic supplementary material.

Supplementary material 1 (DOCX 21 kb)

Supplementary material 2 (DOCX 16 kb)

Supplementary material 3 (DOCX 23 kb)

## Abbreviations

DA: Decision aid
IPDAS: International patient decision aids standards
SDM: Shared decision-making
