# Supplementary material for: Assessing the quality and communicative aspects of patient decision aids for early-stage breast cancer treatment: a systematic review
Source: Breast Cancer Res Treat. 2019 Jul 24;178(1):1–15. doi: 10.1007/s10549-019-05351-4 (PMC6790198; doi:10.1007/s10549-019-05351-4)
Supplement: Supplementary file 1 — Supplementary material 1 (DOCX 21 kb) [file 10549_2019_5351_MOESM1_ESM.docx]

**Supplementary Material 1**

**Table 1.** Search strategy MEDLINE.

| 1 | "Breast Neoplasms"[Mesh] |
| --- | --- |
| 2 | breast*[tiab] AND neoplas*[tiab] |
| 3 | breast*[tiab] AND cancer*[tiab] |
| 4 | breast*[tiab] AND carcin*[tiab] |
| 5 | breast*[tiab] AND tumour*[tiab] |
| 6 | breast*[tiab] AND tumor*[tiab] |
| 7 | breast*[tiab] AND metasta*[tiab] |
| 8 | breast*[tiab] AND malig*[tiab] |
| 9 | "Breast"[Mesh] |
| 10 | neoplas*[tiab] OR cancer*[tiab] OR carcin*[tiab] OR tumo*[tiab] OR metasta*[tiab] OR malig*[tiab] OR "Neoplasms"[Mesh] |
| 11 | #9 AND #10 |
| 12 | #1 OR #2 OR #3 OR #4 OR #5 OR #6 OR #7 OR #8 OR #11 |
| 13 | "Decision Making"[Mesh] |
| 14 | "Clinical Decision-Making"[Mesh] |
| 15 | "Decision Support Systems, Clinical"[Mesh] |
| 16 | "Decision Support Techniques"[Mesh] |
| 17 | "Choice Behavior"[Mesh] |
| 18 | #13 OR #14 OR #15 OR #16 OR #17 |
| 19 | (decision*[tiab] OR decid*[tiab]) AND (support*[tiab] OR tool*[tiab] OR aid*[tiab] OR instrument*[tiab] OR technolog*[tiab] OR system*[tiab]) |
| 20 | decision aid*[tw] |
| 21 | Interactive health communication[tw] |
| 22 | (interacti* AND (internet OR online OR graphic* OR booklet* OR leaflet* OR tool))[tw] |
| 23 | shared decision making[tw] |
| 24 | #19 OR #20 OR #21 OR #22 OR #23 |
| 25 | #18 OR #24 |
| 26 | "Patients"[Mesh] |
| 27 | "Patient Participation"[Mesh] |
| 28 | "Patient Education as Topic"[Mesh] |
| 29 | "Patient Satisfaction"[Mesh] |
| 30 | #26 OR #27 OR #28 OR #29 |
| 31 | #25 OR #30 |
| 32 | “General Surgery”[Mesh] |
| 33 | “Mastectomy”[Mesh] |
| 34 | “Mastectomy, Segmental"[Mesh] |
| 35 | “Mammaplasty”[Mesh] |
| 36 | “Drug Therapy”[Mesh] |
| 37 | “Radiotherapy”[Mesh] |
| 38 | “Radiotherapy, Adjuvant”[Mesh] |
| 39 | #32 OR #33 OR #34 OR #35 OR #36 OR #37 OR #38 |
| 40 | #12 AND #31 AND #39 |
| 41 | Limit 41 to (English or Dutch language and yr=”2006-Current”) |

**Table 2.** Search strategy EMBASE.

| 1 | ‘breast cancer'/exp |
| --- | --- |
| 2 | breast*:ab,ti AND neoplas*:ab,ti |
| 3 | breast*:ab,ti AND cancer*:ab,ti |
| 4 | breast*:ab,ti AND carcin*:ab,ti |
| 5 | breast*:ab,ti AND tumour*:ab,ti |
| 6 | breast*:ab,ti AND tumor*:ab,ti |
| 7 | breast*:ab,ti AND metasta*:ab,ti |
| 8 | breast*:ab,ti AND malig*:ab,ti |
| 9 | ‘breast'/exp |
| 10 | neoplas*:ab,ti OR cancer*:ab,ti OR carcin*:ab,ti OR tumo*:ab,ti OR metasta*:ab,ti OR malig*:ab,ti OR 'neoplasm'/exp |
| 11 | #9 AND #10 |
| 12 | #1 OR #2 OR #3 OR #4 OR #5 OR #6 OR #7 OR #8 OR #11 |
| 13 | ‘decision making’/exp |
| 14 | ‘clinical decision making’/exp |
| 15 | ‘clinical decision support system’/exp |
| 16 | ‘decision support system’/exp |
| 17 | #13 OR #14 OR #15 OR #16 |
| 18 | (decision*:ab,ti OR decid*:ab,ti) AND (support*:ab,ti OR tool*:ab,ti OR aid*:ab,ti OR instrument*:ab,ti OR technolog*:ab,ti OR system*:ab,ti) |
| 19 | decision aid*:ab,ti |
| 20 | Interactive health communication:ab,ti |
| 21 | (interacti* AND (internet OR online OR graphic* OR booklet* OR leaflet* OR tool)):ab,ti |
| 22 | shared decision making:ab,ti |
| 23 | #18 OR #19 OR #20 OR #21 OR #22 |
| 24 | #17 OR #23 |
| 25 | ‘consumer’/exp |
| 26 | ‘patient participation’/exp |
| 27 | ‘patient education’/exp |
| 28 | ‘patient satisfaction’/exp |
| 29 | #25 OR #26 OR #27 OR #28 |
| 30 | #24 OR #29 |
| 31 | ‘breast surgery’/exp |
| 32 | ‘mastectomy’/exp |
| 33 | ‘partial mastectomy’/exp |
| 34 | ‘breast reconstruction’/exp |
| 35 | chemo |
|  | Hormonal |
| 36 | ‘radiotherapy’/exp |
| 37 | ‘adjuvant radiotherapy’/exp |
| 38 | #31 OR #32 OR #33 OR #34 OR #35 OR #36 OR #37 |
| 39 | #12 AND #30 AND #38 |
| 40 | #39 AND ([2006-2018]/py AND ([dutch]/lim OR [english]/lim)) |

**Table 3.** Search strategy CINAHL.

| S1 | (MH "Breast Neoplasms") |
| --- | --- |
| S2 | AB (breast* AND neoplas*) OR TI (breast* AND neoplas*) |
| S3 | AB (breast* AND cancer*) OR TI (breast* AND cancer*) |
| S4 | AB (breast* AND carcin*) OR TI (breast* AND carcin*) |
| S5 | AB (breast* AND tumour*) OR TI (breast* AND tumour*) |
| S6 | AB (breast* AND tumor*) OR TI (breast* AND tumor*) |
| S7 | AB (breast* AND metasta*) OR TI (breast* AND metasta*) |
| S8 | AB (breast* AND malig*) OR TI (breast* AND malig*) |
| S9 | (MH "Breast”) |
| S10 | AB (neoplas* OR cancer* OR carcin* OR tumo* OR metasta* OR malig*) OR TI (neoplas* OR cancer* OR carcin* OR tumo* OR metasta* OR malig*) OR (MS "Neoplasms") |
| S11 | S9 AND S10 |
| S12 | S1 OR S2 OR S3 OR S4 OR S5 OR S6 OR S7 OR S8 OR S11 |
| S13 | (MH “Decision Making”) |
| S14 | (MH “Decision Making, Clinical”) |
| S15 | (MH “Decision Support Systems, Clinical”) |
| S16 | (MH “Decision Support Techniques”) |
| S17 | S13 OR S14 OR S15 OR S16 |
| S18 | AB (decision* OR decid*) AND (support* OR tool* OR aid* OR instrument* OR technolog* OR system*) OR TI (decision* OR decid*) AND (support* OR tool* OR aid* OR instrument* OR technolog* OR system*) |
| S19 | TX decision aid* |
| S20 | TX interactive health communication |
| S21 | TX (interacti* AND (internet OR online OR graphic* OR booklet* OR leaflet* OR tool)) |
| S22 | TX shared decision making |
| S23 | S18 OR S19 OR S20 OR S21 OR S22 |
| S24 | S17 OR S23 |
| S25 | (MH “Patients”) |
| S26 | (MH “Consumer Participation”) |
| S27 | (MH “Patient Education”) |
| S28 | (MH “Patient Satisfaction”) |
| S29 | S25 OR S26 OR S27 OR S28 |
| S30 | S24 OR S29 |
| S31 | (MH “Surgery, Operative+”) |
| S32 | (MH “Mastectomy”) |
| S33 | (MH “Lumpectomy”) |
| S34 | (MH “Breast Reconstruction”) |
| S35 | (MH “Drug Therapy”) |
| S36 | (MH “Radiotherapy”) |
| S37 | (MH “Radiotherapy, Adjuvant”) |
| S38 | S31 OR S32 OR S33 OR S34 OR S35 OR S36 OR S37 |
| S39 | S12 AND S30 AND S38 |
| S40 | S39: Limiters – Published Date: 20060101-20181231; English Language, Dutch Language |

**Table 4.** Search strategy Cochrane Library.

| 1 | MeSH descriptor: [Breast Neoplasms] explode all trees |
| --- | --- |
| 2 | (breast* AND neoplas*):ti, ab, kw (Word variations have been searched) |
| 3 | (breast* AND cancer*):ti, ab, kw (Word variations have been searched) |
| 4 | (breast* AND carcin*):ti, ab, kw (Word variations have been searched) |
| 5 | (breast* AND tumour*):ti, ab, kw (Word variations have been searched) |
| 6 | (breast* AND tumor*):ti, ab, kw (Word variations have been searched) |
| 7 | (breast* AND metasta*):ti, ab, kw (Word variations have been searched) |
| 8 | (breast* AND malig*):ti, ab, kw (Word variations have been searched) |
| 9 | MeSH descriptor: [Breast] explode all trees |
| 10 | MeSH descriptor: [Neoplasms] explode all trees |
| 11 | (neoplas* OR cancer* OR carcin* OR tumo* OR metasta* OR malig*):ti, ab, kw (Word variations have been searched) |
| 12 | #9 AND (#10 OR #11) |
| 13 | #1 OR #2 OR #3 OR #4 OR #5 OR #6 OR #7 OR #8 OR #12 |
| 14 | MeSH descriptor: [Decision Making] this term only |
| 15 | MeSH descriptor: [Clinical Decision-Making] this term only |
| 16 | MeSH descriptor: [Decision Support Systems, Clinical] this term only |
| 17 | MeSH descriptor: [Decision Support Techniques] this term only |
| 18 | MeSH descriptor: [Choice Behavior] this term only |
| 19 | #14 OR #15 OR #16 OR #17 OR #18 |
| 20 | (decision* OR decid*) AND (support* OR tool* OR aid* OR instrument* OR technolog* OR system*):ti, ab, kw (Word variations have been searched) |
| 21 | decision aid:ti, ab, kw (Word variations have been searched) |
| 22 | Interactive health communication:ti, ab, kw (Word variations have been searched) |
| 23 | (interacti* AND (internet OR online OR graphic* OR booklet* OR leaflet* OR tool)) :ti, ab, kw (Word variations have been searched) |
| 24 | shared decision making:ti, ab, kw (Word variations have been searched) |
| 25 | #20 OR #21 OR #22 OR #23 OR #24 |
| 26 | #19 OR #25 |
| 27 | MeSH descriptor: [Patients] explode all trees |
| 28 | MeSH descriptor: [Patient Participation] this term only |
| 29 | MeSH descriptor: [Patient Education as Topic] this term only |
| 30 | MeSH descriptor: [Patient Satisfaction] this term only |
| 31 | #27 OR #28 OR #29 OR #30 |
| 32 | #26 OR #31 |
| 33 | MeSH descriptor: [Surgical Procedures, Operative] this term only |
| 34 | MeSH descriptor: [Mastectomy] explode all trees |
| 35 | MeSH descriptor: [Mammaplasty] explode all trees |
| 36 | MeSH descriptor: [Drug Therapy] explode all trees |
| 37 | MeSH descriptor: [Radiotherapy] this term only |
| 38 | MeSH descriptor: [Radiotherapy, Adjuvant] this term only |
| 39 | #33 OR #34 OR #35 OR #36 OR #37 OR #38 |
| 40 | #13 AND #32 AND #39 |
| 41 | #40 in Trials |
| 42 | Limit #41 to (yr=”2006-Current”) |

**Table 5.** Search strategy PsycINFO.

| 1 | exp Breast Neoplasms/ |
| --- | --- |
| 2 | (breast* AND neoplas*).ti,ab. |
| 3 | (breast* AND cancer*).ti,ab. |
| 4 | (breast* AND carcin*).ti,ab. |
| 5 | (breast* AND tumour*).ti,ab. |
| 6 | (breast* AND tumor*).ti,ab. |
| 7 | (breast* AND metasta*).ti,ab. |
| 8 | (breast* AND malig*).ti,ab. |
| 9 | exp Breast/ |
| 10 | (neoplas* OR cancer* OR carcin* OR tumo* OR metasta* OR malig*).ti,ab. OR (exp Neoplasms/) |
| 11 | #9 AND #10 |
| 12 | #1 OR #2 OR #3 OR #4 OR #5 OR #6 OR #7 OR #8 OR #11 |
| 13 | exp Decision Making/ |
| 14 | Clinical decision-making.mp |
| 15 | exp Decision Support Systems/ |
| 16 | Decision support techniques.mp |
| 17 | exp Choice Behavior/ |
| 18 | #13 OR #14 OR #15 OR #16 OR #17 |
| 19 | (decision* OR decid*) AND (support* OR tool* OR aid* OR instrument* OR technolog* OR system*).mp |
| 20 | decision aid*.mp |
| 21 | Interactive health communication.mp |
| 22 | (interacti* AND (internet OR online OR graphic* OR booklet* OR leaflet* OR tool)).mp |
| 23 | shared decision making.mp |
| 24 | #19 OR #20 OR #21 OR #22 OR #23 |
| 25 | #18 OR #24 |
| 26 | exp patients/ |
| 27 | exp client participation/ |
| 28 | exp client education/ |
| 29 | exp client satisfaction/ |
| 30 | #26 OR #27 OR #28 OR #29 |
| 31 | #25 OR #30 |
| 32 | exp surgery/ |
| 33 | exp mastectomy/ |
| 34 | Breast-conserving.mp |
| 35 | breast reconstruction.mp |
| 36 | exp Drug Therapy/ |
| 37 | exp Radiation Therapy |
| 38 | #32 OR #33 OR #34 OR #35 OR #36 OR #37 |
| 39 | #12 AND #31 AND #38 |
| 40 | Limit 47 to (english language and dutch language and yr=”2006-Current”) |
