# Supplementary material for: Assessing the quality and communicative aspects of patient decision aids for early-stage breast cancer treatment: a systematic review
Source: Breast Cancer Res Treat. 2019 Jul 24;178(1):1–15. doi: 10.1007/s10549-019-05351-4 (PMC6790198; doi:10.1007/s10549-019-05351-4)
Supplement: Supplementary file 2 — Supplementary material 2 (DOCX 16 kb) [file 10549_2019_5351_MOESM2_ESM.docx]

**Supplementary Material 2**

**Table 1.** Results from the International Patient Decision Aids Standards (IPDAS) Checklist of the Patient Decision Aids (*n*=21).

| **Item** | **IPDAS dimension** | **Item description** | ***n*** | **%** |
| --- | --- | --- | --- | --- |
| 1 | Information about options | The DST describes the health condition or problem (intervention, procedure, or investigation) for which the index decision is required | 20 | 95 |
| 2 |  | The DST described the decision that needs to be considered (the index decision) | 21 | 100 |
| 3 |  | The DST describes the options available for the index decision | 21 | 100 |
| 4 |  | The DST describes the natural course of the health condition or problem, if no action is taken | 16 | 76 |
| 5 |  | The DST describes positive features (benefits or advantages) of each option | 20 | 95 |
| 6 |  | The DST describes negative features (harms, side effects or disadvantages) of each option | 20 | 95 |
| 7 |  | The DST makes it possible to compare the positive and negative features of the available options | 18 | 86 |
| 8 |  | The DST shows the negative and positive features of options with equal detail | 3 | 14 |
| 9 | Outcome probabilities | The DST provides information about outcome probabilities associated with the options (i.e, the likely consequences of decisions) | 16 | 76 |
| 10 |  | The DST specifies the defined group (reference class) of patients for which the outcome probabilities apply | 12 | 57 |
| 11 |  | The DST specifies the event rates for the outcome probabilities | 12 | 57 |
| 12 |  | The DST specifies the time period over which the outcome probabilities apply | 9 | 43 |
| 13 |  | The DST allows the user to compare outcome probabilities across options using the same denominator and time period | 6 | 29 |
| 14 |  | The DST provides information about the levels of uncertainty around event or outcome probabilities | 11 | 52 |
| 15 |  | The DST provides more than one way of viewing the probabilities | 10 | 49 |
| 16 |  | The DST provides balanced information about event or outcome probabilities to limit framing bias | 9 | 43 |
| 17 | Clarifying values | The DST describes the features of options to help patients imagine what it is like to experience physical effects | 18 | 86 |
| 18 |  | The DST describes the features of options to help patients imagine what it is like to experience the psychological effects | 16 | 76 |
| 19 |  | The DST describes the features of options to help patients imagine what it is like to experience social effects | 14 | 67 |
| 20 |  | The DST asks patients to think about which positive and negative features of the options matters most to them | 21 | 100 |
| 21 | Decision guidance | The DST provides a step-by-step way to make a decision | 17 | 81 |
| 22 |  | The DST includes tools like worksheets or lists of questions to use when discussing options with a practitioner | 17 | 81 |
| 23 | Development process | The DST (or associated paper) mentions that the development process included finding out what clients or patients need to prepare them to discuss a decision | 12 | 57 |
| 24 |  | The DST (or associated paper) mentions that the development process included finding out what health professionals need to prepare them to discuss a specific decision with patients | 9 | 43 |
| 25 |  | The DST (or associated paper) mentions that the development process included expert review by clients/patients not involved in producing the DST | 11 | 52 |
| 26 |  | The DST (or associated paper) mentions that the development process included expert review by health professionals not involved in producing the DST | 20 | 95 |
| 27 |  | The DST (or associated paper) mentions that the DST was field tested with patients who were facing the decision | 12 | 57 |
| 28 |  | The DST (or associated paper) mentions that the DST was field tested with practitioners who counsel patients who face the decision | 12 | 57 |
| 29 | Using evidence | The DST (or associated paper) provides citations to the studies selected | 13 | 62 |
| 30 |  | The DST (or associated paper) describes how research evidence was selected or synthesized | 10 | 48 |
| 31 |  | The DST (or associated paper) provides a production or publication rate | 17 | 81 |
| 32 |  | The DST (or associated paper) provides information about the proposed update policy | 4 | 19 |
| 33 |  | The DST (or associated paper) describes the quality of the research evidence used | 3 | 14 |
| 34 | Disclosure and transparency | The DST (or associated technical documentation) provides information about the funding used for development | 15 | 71 |
| 35 |  | The DST includes author / developer credentials or qualifications | 19 | 90 |
| 36 | Plain language | The DST (or associated paper) reports readability levels (using one or more of the available scales) | 5 | 24 |

*Note.* DST = Decision support technology.
