# Supplementary material for: Assessing the quality and communicative aspects of patient decision aids for early-stage breast cancer treatment: a systematic review
Source: Breast Cancer Res Treat. 2019 Jul 24;178(1):1–15. doi: 10.1007/s10549-019-05351-4 (PMC6790198; doi:10.1007/s10549-019-05351-4)
Supplement: Supplementary file 3 — Supplementary material 3 (DOCX 23 kb) [file 10549_2019_5351_MOESM3_ESM.docx]

**Supplementary Material 3**

**Table 1.** Results from the Communicative Aspects (CAs) Checklist of the Patient Decision Aids (*n*=21).

| **Item** | **Aspect** | **Item description** | ***n*** | **%** |
| --- | --- | --- | --- | --- |
| 1 | Information presentation | Number of decision aids that included probabilistic information | 18 | 86 |
|  |  | Methods used to communicate probabilistic information (*n*=18) |  |  |
| 2 |  | Verbal |  |  |
|  |  | Absolute risks descriptions | 18 | 100 |
|  |  | Relative risks descriptions | 12 | 67 |
| 3 |  | Numerical (*n* = 16) |  |  |
|  |  | Percentages | 11 | 69 |
|  |  | Natural frequencies | 12 | 75 |
|  |  | Absolute risks | 13 | 81 |
|  |  | Relative risks | 2 | 13 |
|  |  | Absolute risk reduction | 1 | 6 |
|  |  | Relative risk reduction | 2 | 13 |
|  |  | Number needed to treat/harm | 0 | 0 |
| 4 |  | Visual (*n* = 10) |  |  |
|  |  | Pie chart | 1 | 10 |
|  |  | Bar chart | 0 | 0 |
|  |  | Line graph | 1 | 10 |
|  |  | Icon array | 9 | 90 |
|  |  | Risk scale | 0 | 0 |
| 5 |  | Number of decision aids that described uncertainties around probabilities (*n*=18) | 14 | 78 |
|  |  | Methods used to communicate uncertainties (*n*=14): |  |  |
| 6 |  | Verbal |  |  |
|  |  | Textual descriptions | 13 | 93 |
| 7 |  | Numerical |  |  |
|  |  | Numerical range | 8 | 57 |
| 8 |  | Visual |  |  |
|  |  | Confidence intervals | 1 | 7 |
|  |  | Colored pictograms | 0 | 0 |
| 9 |  | Number of decision aids that included disease-related information | 15 | 71 |
|  |  | Methods used to communicate this information (*n*=15): |  |  |
| 10 |  | Verbal (text) | 15 | 100 |
| 11 |  | Visual (illustrations) | 9 | 60 |
| 12^a^ |  | Audiovisual (video clips) (*n*=8) | 1 | 13 |
| 13^a^ |  | Audio (audio clips) (*n*=8) | 1 | 13 |
| 14 |  | Number of decision aids that included information about procedures of treatments | 21 | 100 |
|  |  | Methods used to communicate this information: |  |  |
| 15 |  | Verbal (text) | 21 | 100 |
| 16 |  | Visual (illustrations) | 14 | 67 |
| 17^a^ |  | Audiovisual (video clips) (*n*=11) | 4 | 36 |
| 18^a^ |  | Audio (audio clips) (*n*=11) | 1 | 9 |
| 19 |  | Number of decision aids that presented the information in a balanced and unbiased way | 2 | 10 |
|  |  | Methods used for balanced and unbiased information: |  |  |
| 20 |  | Uses roughly the same amount of text for each option | 12 | 57 |
| 21 |  | Displays statistics in the same way for each option (*n*=16) | 5 | 31 |
| 22 |  | Uses similar fonts for each option | 21 | 100 |
| 23 |  | Uses language that is not biased in favor of a specific option | 16 | 76 |
| 24 |  | Presents equal number of positive features of each option (*n*=20) | 9 | 45 |
| 25 |  | Presents equal number of negative features of each option (*n*=20) | 4 | 20 |
| 26 |  | Keeps the order of positive and negative features constant (*n*=20) | 17 | 85 |
| 27 | Information control | The decision aid allows for patients to only receive information that they want to read | 9 | 43 |
| 28 |  | The decision aid provides a step-by-step way to move through the decision aid | 18 | 86 |
| 29 |  | The decision aid provides the patient the opportunity to read more about a specific topic of interest | 16 | 76 |
| 30 |  | The decision aid provides access to external sources | 16 | 76 |
| 31 |  | The decision aid provides access to internal sources | 11 | 52 |
| 32 |  | The decision aid allows for patients to search for key words | 5 | 24 |
| 33^a^ |  | The decision aid makes it easy for patients to return to previous parts of the decision aid (*n*=11) | 11 | 100 |
| 34 | Personalized information | Tailoring in general towards type of treatment | 7 | 33 |
| 35 |  | Tailoring in general towards specific populations | 3 | 14 |
| 36 |  | Tailoring in general towards disease factors | 4 | 19 |
| 37 |  | Tailoring in general towards breast cancer stage | 14 | 67 |
| 38 |  | Probability tailoring | 2 | 10 |
| 39 |  | Content tailoring | 5 | 24 |
| 40 |  | Mode of presentation tailoring | 3 | 14 |
| 41 | Interaction | Number of decision aids that help patients to consider personal values and preferences | 20 | 95 |
|  |  | Methods used to consider or assess values and preferences (*n*=20): |  |  |
| 42 |  | Passive |  |  |
|  |  | Asks patients to think about their values and preferences | 20 | 100 |
|  |  | Active |  |  |
| 43 |  | Weighting exercises | 12 | 60 |
| 44 |  | Sliders to assign values to preferences | 9 | 45 |
| 45 |  | Number of decision aids that help allow for comparison of positive and negative features of treatment options | 20 | 21 |
|  |  | Methods used to compare positive and negative features of options (*n*=20): |  |  |
| 46 |  | Ranking or rating scale | 6 | 30 |
| 47 |  | Table to compare positive and negative features | 17 | 85 |
| 48 |  | Verbal comparisons | 18 | 90 |
| 49 |  | Conjoint analysis / Visual analogue scale | 2 | 10 |
| 50 |  | Number of decision aids that provide patient the most suitable treatment option | 3 | 14 |
|  |  | Methods used to provide feedback: |  |  |
| 51 |  | The decision aid shows the progress of the decision aid | 12 | 57 |
| 52 |  | The decision aid provides patients a summary of their values and lll preferences | 12 | 57 |
| 53 |  | The decision aid permits printing as a single document | 17 | 81 |
| 54 |  | The decision aid provides space for note taking | 10 | 48 |
| 55 |  | The decision aid includes a short knowledge test | 8 | 38 |
| 56 | Accessibility of information | The decision aid is freely available on the web | 16 | 76 |
| 57 |  | The decision aid requires a login code | 5 | 24 |
| 58 |  | The decision aid is purely computer based | 11 | 52 |
| 59 |  | The decision aid requires access to internet for its use | 11 | 52 |
| 60 |  | The decision aid reports last update | 13 | 62 |
| 61 |  | The decision aid reports update frequency | 2 | 10 |
| 62 |  | The decision aid requires staff assistance | 6 | 29 |
| 63 |  | The decision aid is self-administered | 20 | 95 |
| 64 |  | The decision aid can be used on multiple devices | 20 | 95 |
| 65 | Suitability of information | The decision aid contains less than 10 (web) pages | 5 | 24 |
| 66^a^ |  | The decision aid contains videos with a length of less than 1 minute (*n*=6) | 1 | 17 |
| 67 |  | The decision aid has a conversational (writing) style | 19 | 90 |
| 68 |  | The decision aid has irrelevant illustrations | 6 | 29 |
| 69 | Source of information | Number of decision aids that mentioned on which datasets the probabilistic information are based on (*n*=18) | 5 | 28 |
|  |  | Types of datasets (*n*=5): |  |  |
|  |  | Observational data | 2 | 40 |
|  |  | Randomized controlled trials data | 1 | 20 |
|  |  | Patient reported outcomes data | 1 | 20 |
|  |  | Data combined from different studies | 1 | 20 |
|  |  | Types of outcome probabilities reported by the decision aid (*n*=18): |  |  |
| 70 |  | Mortality rate / Survival rate | 5 | 28 |
| 71 |  | Incidence rate / Progression free survival | 12 | 67 |
| 72 |  | Treatment side effects | 12 | 67 |
| 73 |  | Quality of life | 4 | 22 |
|  |  | Type of information about the data(sets) provided by the decision aid (*n=*5): |  |  |
| 74 |  | About what scale the patient data have been collected | 1 | 20 |
| 75 |  | About the number of patients on which the data are based on | 3 | 60 |
|  |  | About characteristics of patients on which the data are based on | 3 | 60 |
| 76 |  | About the period of time of data collection |  |  |

*Note.* ^a^ This item does not apply to paper-based decision aids.
